# Clinical impact of TP53 disruption in chronic lymphocytic leukemia patients treated with ibrutinib: a campus CLL study

**DOI:** 10.1038/s41375-023-01845-9

**Published:** 2023-02-18

**Authors:** Riccardo Bomben, Francesca Maria Rossi, Filippo Vit, Tamara Bittolo, Antonella Zucchetto, Robel Papotti, Erika Tissino, Federico Pozzo, Massimo Degan, Jerry Polesel, Pietro Bulian, Roberto Marasca, Gianluigi Reda, Luca Laurenti, Jacopo Olivieri, Annalisa Chiarenza, Roberta Laureana, Massimiliano Postorino, Maria Ilaria Del Principe, Antonio Cuneo, Massimo Gentile, Fortunato Morabito, Gilberto Fronza, Agostino Tafuri, Francesco Zaja, Robin Foà, Francesco Di Raimondo, Giovanni Del Poeta, Valter Gattei

**Affiliations:** 1 Clinical and Experimental Onco-Haematology Unit, Centro di Riferimento Oncologico di Aviano (CRO) IRCCS, Aviano, Italy; 2 International PhD School in Clinical and Experimental Medicine, University of Modena and Reggio Emilia, Modena, Italy; 3 Unit of Cancer Epidemiology, Centro di Riferimento Oncologico di Aviano (CRO) IRCCS, Aviano, Italy; 4 Hematology Unit, Department of Oncology and Hematology, Azienda-Ospedaliero Universitaria (AOU) of Modena, Policlinico, Modena, Italy; 5 Department of Medical and Surgical Sciences, University of Modena e Reggio Emilia, Modena, Italy; 6 Division of Ematologia, Fondazione IRCCS Ca’Granda Ospedale Maggiore Policlinico di Milano, Milano, Italy; 7 Fondazione Universitaria Policlinico A Gemelli di Roma, Roma, Italy; 8 Clinica Ematologica, Centro Trapianti e Terapie Cellulari “Carlo Melzi” DISM, Azienda Ospedaliera Universitaria S. Maria Misericordia, Udine, Italy; 9 Division of Hematology, Policlinico, Department of Surgery and Medical Specialties, University of Catania, Catania, Italy; 10 Division of Haematology, University of Tor Vergata, Rome, Italy; 11 Hematology Section, Department of Medical Sciences, University of Ferrara, Ferrara, Italy; 12 Hematology Unit AO of Cosenza, Cosenza, Italy; 13 Department of Pharmacy, Health and Nutritional Sciences, University of Calabria, Rende, Italy; 14 Hematology Oncology Department, Augusta Victoria Hospital, East Jerusalem, Israel; 15 Mutagenesis and Cancer Prevention Unit, IRCCS Ospedale Policlinico San Martino, Genoa, Italy; 16 Department of Clinical and Molecular Medicine and Hematology, Sant’Andrea - University Hospital - Sapienza, University of Rome, Rome, Italy; 17 Department of Medical, Surgical and Health Sciences, University of Trieste, Trieste, Italy; 18 Hematology, Department of Translational and Precision Medicine, ‘Sapienza’ University, Rome, Italy; 19 Present Address: Clinical and Experimental Onco-Haematology Unit, Centro di Riferimento Oncologico di Aviano (CRO) IRCCS, Aviano, Italy

**Keywords:** Risk factors, Preclinical research

## To the Editor

Disruption of the *TP53* gene, either by deletion at chromosome 17p13.1 (del17p) or mutations, is the most important prognostic/predictive biomarker in chronic lymphocytic leukemia (CLL), also in the context of the novel target therapies including ibrutinib [1–4]. Although *TP53* deletion and mutations mostly co-occur and are considered as equal prognosticators, the prognostic value of isolated or concomitant mutations and deletions remains unclear [2, 3]. Here we applied an ultra-deep next-generation sequencing (NGS) approach in CLL patients treated with ibrutinib, to investigate the clinical impact of *TP53* mutations and del17p, either concomitant or isolated, or in relation to their disruption burden.

This study, generated in the framework of an institutional Italian multicenter working group on CLL (“Campus CLL”), is a retrospective/multicenter analysis of 229 CLL patients treated with ibrutinib in the current clinical practice. All cases have been either referred to a single institution for molecular and cytogenetic analyses (February 2014–February 2021), or retrospectively referred by delivering frozen cell samples taken prior to starting ibrutinib treatment. Clinical outcome data were updated as of October 2021. Eighty patients, included in a previous study [3], are presented here with an updated median follow-up (24.7 months). As a stringent criterion, only patients assayed for *TP53* mutation and 17p deletion in the same blood sample taken within 6 months prior to the start of ibrutinib were included. Median follow-up from ibrutinib treatment was 36.3 months (95% CI 29.5–41.5 months); 51 patients were treatment naïve (TN) and, 178 refractory/relapsed (RR). In accordance with the ERIC recommendations for *TP53* disruption [5], mutation analyses were always carried out on samples containing >80% tumor cells; when lower than the 80% cutoff, CD19 positive CLL cells were purified by cell sorting. Briefly, analysis of *TP53* mutations was performed with an amplicon-based strategy, covering exons 2–11, as previously reported [4]. A minimum coverage of 2,000X was obtained for each sequence in 100% of the analyzed positions, with a limit of detection of 0.3% VAF; *TP53* mutated cases with less than 2% VAF were all confirmed by a second independent NGS run starting from DNA [4]. Moreover, selected low-VAF *TP53* mutations were verified by a different experimental approach (digital droplet PCR, ddPCR). *BTK* and *PLCG2* mutations related to ibrutinib resistance were studied by NGS. Interphase FISH was performed to detect del17p and 11q22.3 deletion (del11q) [4]. Further methodological details are provided in Supplementary Information. The clinical and biological baseline characteristics of patients [6] are detailed in Supplementry Table S1. All statistical analyses were performed by using standard methods. Overall survival (OS) and progression free survival (PFS) were computed from date of ibrutinib treatment to date of death or progression/suspension (events), respectively, or last follow-up (censoring). Molecular studies were blinded to the study end points.

Among 229 patients, 68 died and 57 progressed after median follow-up of 15.6 months (95% CI 11.9–20.5 months) and 24 months (95% CI 16.0–32.7 months) from ibrutinib starting, respectively. As in previous reports [7–9], Rai stage, the number of previous treatments (0/1 versus >1), anemia and abnormal LDH values were found to associate with shorter PFS and/or OS by univariable analyses (Table 1 and Supplementry Fig. S1).

**Table 1:** Univariable and multivariable analyses of OS and PFS (*n* = 229).

	UVA				MVA^a^				Bootstrap selction (%)^g^	UVA				MVA^b^				Bootstrap selction (%)^g^
	HR	LCI	UCI	*P*	HR	LCI	UCI	*P*		HR	LCI	UCI	*P*	HR	LCI	UCI	*P*	
Gender (Male)	1.47	0.86	2.51	0.1644	–				27	1.73	1.08	2.78	**0.0233**	ni				47
Age (≥65 y)	1.18	0.69	2.00	0.5489	–				7	1.03	0.66	1.61	0.8928	–				4
Rai stage (I-II versus III-IV)^c^	1.82	1.12	2.94	**0.0147**	ni				11	1.65	1.08	2.51	**0.0201**	ni				17
Previous Line of therapy (0–1 versus >1)	1.93	1.19	3.12	**0.0077**	1.91	1.17	3.13	**0.0093**	69	2.34	1.54	3.55	**<0.0001**	2.44	1.59	3.74	**<0.0001**	96
Anemia	2.86	1.77	4.62	**<0.0001**	2.47	1.51	4.05	**0.0003**	83	2.25	1.49	3.41	**0.0001**	2.04	1.33	3.13	**0.0011**	65
β2 microglobulin (high)^d^	1.68	0.96	2.95	0.0689	–					1.61	0.99	2.61	0.0526	–				
LDH (high)^e^	1.61	0.98	2.65	0.0607	ni				11	1.74	1.14	2.67	**0.0108**	ni				7
IGHV (UM)	1.49	0.83	2.68	0.1849	–				15	1.81	1.07	3.08	**0.0272**	ni				36
del11q (present)^d^	1.31	0.77	2.22	0.3233	–				39	1.21	0.76	1.93	0.4199	–				28
del17p/non-*TP53*mut^f^	0.60	0.08	4.50	0.6208	0.71	0.09	5.35	0.7391	2	0.33	0.05	2.42	0.2756	0.34	0.05	2.53	0.2942	20
Non-del17p/TP53mut^f^	1.46	0.77	2.76	0.2467	1.43	0.74	2.79	0.2917	26	1.02	0.58	1.77	0.9556	0.90	0.51	1.61	0.7340	8
del17p/TP53mut^f^	2.16	1.21	3.85	**0.009**	2.27	1.24	4.14	**0.0077**	81	2.01	1.24	3.25	**0.0047**	2.05	1.24	3.37	**0.0049**	70

*Anemia* <110 g/L for women or <120 g/L for men, *IGHV unmutated (UM)* ≥98% identity with germ line, *β2 microglobulin and LDH high* >upper normal level according to the different laboratories, *OS* overall survival from ibrutinib start, *PFS* progression free survival, *UVA* univariable analysis, *MVA* multivariable analysis, *HR* Hazard Ratio, *CI* confidence interval, *LCI* 95% lower CI, *UCI* 95% Upper CI, – not used in the final model, *ni* not included in the final model.

^a^Multivariable analysis was carried out using the following variables (*n* = 219): Rai stage, previous lines of therapies, anemia, LDH, *TP53* disruptions.

^b^Multivariable analysis was carried out using the following variables (*n* = 219): gender, Rai stage, previous lines of therapies, anemia, LDH, IGHV, *TP53* disruptions.

^c^Available for 221 patients.

^d^Available for 196 patients.

^e^Available for 227 patients.

^f^Treated as categorical variables respect to wt cases.

^g^Internal bootstrapping validation; figures reported the percentage (rounded to unity) of *P* < 0.05 in 500 replications; β2 microglobulin was excluded from bootstrapping validation.

Bold values indicate statistical significance *p* < 0.05.

**Fig. 1:**
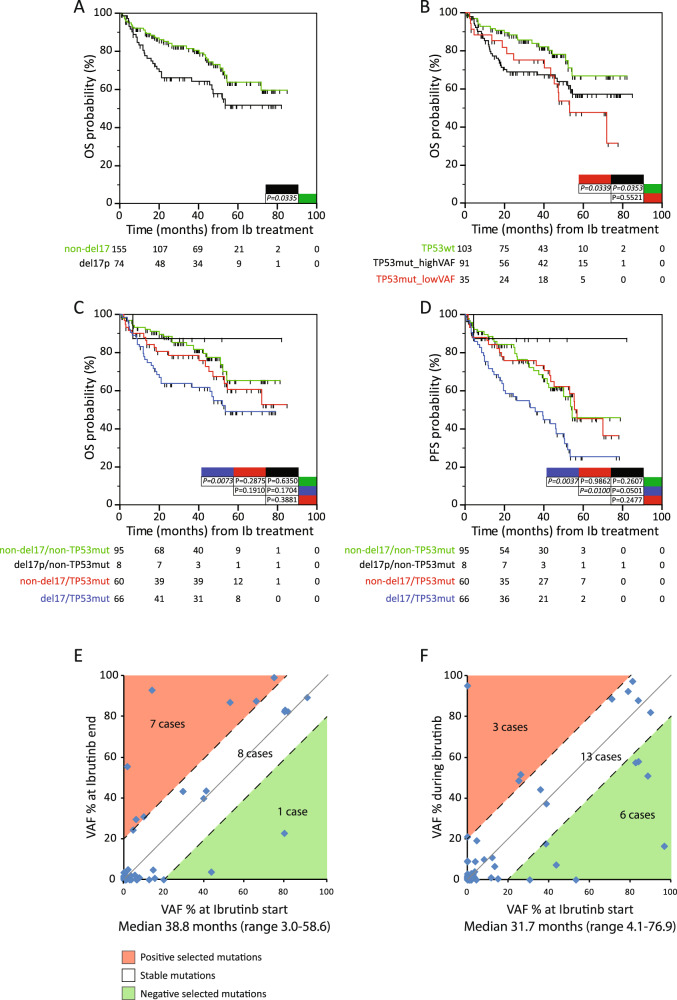
Clinical impact of *TP53* aberrations in ibrutinib-treated CLL. **A** Kaplan–Meier curves comparing OS probabilities of 155 non-del17p cases (green line), 74 cases with del17p (black line). **B** Kaplan–Meier curves comparing OS probabilities of 103 *TP53*wt cases (green line), 91 cases with high-VAF *TP53* mutations (TP53mut_highVAF), i.e., ≥10.0% of VAF (black line), and 35 cases with low-VAF *TP53* mutations (TP53mut_lowVAF), i.e., <10.0% of VAF (red line). **C** Kaplan–Meier curves comparing OS probabilities of 95 cases lacking del17p and *TP53* mutations (non-del17p/non-*TP53*mut, green line), 8 cases with del17p only (del17p/non-TP53mut, black line), 60 cases with *TP53* mutations only (non-del17p/TP53mut, red line), and 66 cases with concomitant *TP53* mutation and del17p (del17p/TP53mut, blue line). **D** Kaplan–Meier curves comparing PFS probabilities of 95 cases lacking del17p and *TP53* mutations (non-del17p/non-*TP53*mut, green line), 8 cases with del17p only (del17p/non-TP53mut, black line), 60 cases with *TP53* mutations only (non-del17p/TP53mut, red line), and 66 cases with a concomitant *TP53* mutation and del17p (del17p/TP53mut, blue line). In Kaplan–Meier curves, cases with more than one mutation are classified according to the mutation with the highest VAF (see Supplementry Table S3). The number of patients in each group is reported; *P* values refer to the log-rank test. **E** Clonal evolution of *TP53* mutations in longitudinal samples relapsed under ibrutinib treatment (relapsed cases). Graph reports results for 16 CLL patients (65 *TP53* mutations) longitudinally studied at two different time-points, the 1^st^ (x-axis) collected at ibrutinib start and the 2^nd^ (y-axis) collected after ibrutinib interruption for relapse; overall, VAF values are referred to 65 *TP53* mutations, as measured at the two time-points. **F** Clonal evolution of *TP53* mutations in longitudinal samples during ibrutinib treatment (non-relapsed cases). Graph reports results for 22 CLL patients (62 *TP53* mutations) longitudinally studied at two different time-points, the 1^st^ (x-axis) collected at ibrutinib start and the 2^nd^ (y-axis) collected during ibrutinib treatment; overall, VAF values are referred to 62 *TP53* mutations, as measured at the two time-points. The red color denotes a mutation with a VAF increase greater than 20%. The green color denotes a mutation with a VAF decrease greater than 20%. The dotted parallel lines denote the 20% interval on either side.

CLL bearing del17p (*n* = 74; Supplementry Table S1) showed inferior OS and PFS compared to non-del17p cases (Fig. 1A and Supplementry Table S2), as previously reported [10]. Consistently, del17p was independent predictor in multivariable models for OS/PFS (*P* = *0.0209*, OS; *P* = *0.0057*, PFS; Model 1 Supplementry Table S2). At baseline, before ibrutinib treatment, we identified a total of 296 *TP53* mutations in 126 patients (median mutations per patient: 1; range of mutations/patient: 1–11; Supplementry Table S3). The relative high proportion of cases (126/229, 55%) with *TP53* mutations can be explained by the use of an ultra-deep NGS strategy that allows the detection of very small mutated clone (see also Supplementry Table S4 and Supplementry Fig. S2 for ddPCR validation of selected mutations) [4, 5]. By classifying *TP53*-mutated patients according to the VAF of the most prevalent *TP53* mutation, VAF range for *TP53*-mutated cases was 0.53–95.24% (Supplementry Table S3). As in the chemo-immuno therapy setting [4], also in the ibrutinib setting, patients bearing *TP53* mutations with low (<10%) and high (≥10%) VAF had shorter OS than *TP53*wt cases, either kept separate (Fig. 1B), or when low-VAF and high-VAF cases were combined (Supplementry Fig. S3). These results suggest that even low burden *TP53* alterations confer a negative impact on outcomes, widening previous findings [11]. Accordingly, *TP53* mutations were associated with shorter OS/PFS intervals in univariable analyses (Supplementry Table S2), as well as in an OS multivariable model (*P* = *0.0217*; Model 2, Supplementry Table S2). Here, we expanded to low-VAF *TP53*-mutated patients previous observations on the clinical impact of *TP53* disruption upon ibrutinib, as they emerged in the context of clinical trials [7], or in real-life [3, 6, 8], where *TP53* disrupted patients were identified according to the current standard criteria (i.e. VAF ≥ 10%).

The combination of del17p with *TP53* mutation data identified 95 cases without any *TP53* aberrations (non-del17p/non-*TP53*mut), 8 del17p only cases, 60 *TP53*-mutated only cases (28 low-VAF), and 66 cases bearing both del17p deletion and *TP53* mutations (7 low-VAF). Only patients with concomitant *TP53* mutations and del17p showed significantly shorter OS/PFS intervals compared to non-del17p/non-*TP53*mut cases, while no difference in OS/PFS was found in patients presenting single aberration (Fig. 1C, D). The simultaneous presence of *TP53* mutations and del17p confirmed its detrimental clinical impact by univariable analysis and remained independent predictor for short OS/PFS by multivariable analyses together with the number of previous lines of therapy and anemia; consistently, these variables were the most frequently selected by internal bootstrap validation (Table 1). Given the low number of patients of some subgroups (e.g. 8 del17p alone cases), these results need to be confirmed in larger cohorts.

At variance from chemo-immunotherapy where the presence of a single *TP53* mutation, even with a low-VAF, is associated with a worse outcome [4, 12], in the ibrutinib setting only cases presenting a more complex disruption of the TP53 function, due to the concomitant presence of mutations and deletions, fail to have the best benefit from therapy. Our results are in keeping with recent findings suggesting that only double-hit aberrations (i.e. more than one *TP53* mutation or *TP53* mutation and del17p) are independently associated with a shorter outcome in ibrutinib-treated patients, single-hit aberrations (a single *TP53* mutation or del17p only) having an outcome comparable to that of *TP53*wt patients [2]. Differently from Brieghel et al. [2], however, in our cohort, *TP53* mutated patients with more than one mutations but without del17p failed to experience a significantly worse prognosis respect to patients without any aberrations (data not shown). In the present series, 52/66 cases concomitantly bearing del17p and *TP53* mutations (79%) bore *TP53* mutations and/or 17p deletion in most of the neoplastic clone (Supplementry Table S3). We could, therefore, speculate that the genetic instability fostered by such a massive *TP53* disruption might eventually lead to the development of more complex genetic lesions, known to correlate with dismal outcomes in the ibrutinib setting [11, 13]. Our finding may help to explain previous reports of ibrutinib-treated CLL in which *TP53* mutations failed to have a prognostic impact [12], and in which the simultaneous presence of *TP53* mutations and deletion was not investigated.

The evolution of *TP53* mutated clones was assessed in 38 patients by longitudinal NGS analysis of paired samples collected at pre-treatment (median time, −0.9 month; range −6.0–0.0) and during (non-relapsed cases; *n* = 22) or after (relapsed cases; *n* = 16) ibrutinib treatment (median time interval, 31.8 months, range 3.0–76.9). For relapsed cases, the second time point was collected in close proximity of progression (median time, −0.7 months, range −3.0–1.0 months). No significant differences were observed between relapsed and non-relapsed cases in relation to the timing of the second sampling (*P* = 0.74). Of a total of 127 *TP53* mutations, 92 were present before and 106 after treatment; among these, 21 mutations (median VAF, 1.7%, range 0.4–52.3%) disappeared during the course of treatment, while 35 were newly identified (median VAF, 1.0%, range 0.4–95.2%; Supplementry Table S5). Among relapsed cases, 15/16 showed either a prominent expansion (i.e. a VAF increase greater than 20%) or stability (i.e. VAF variations within the range of 20% VAF variation) over time of the *TP53* mutated clone(s) (Fig. 1E). Conversely, in the context of non-relapsed patients, 3 cases presented a VAF increase of the *TP53* mutated clones, 13 remained stable, and 6 showed a VAF reduction (Fig. 1F). These data support the idea of a general stability of *TP53* subclones under ibrutinib [14], although a positive selection of *TP53* mutations over time was slightly over-represented in relapsed cases (*P* = 0.04, χ^2^ test), suggesting the occurrence of other genetic events complementing the clonal advantage due to *TP53* disruption [11, 14, 15]. Considering the 127 *TP53* mutations identified across the different time-points, 8 mutations were shared by ≥3 cases (Supplementry Table S5). Among them, G245S and R175H were found expanded (>20% VAF increase) in 3/4 and 2/3 cases, respectively (Supplementry Table S5), suggesting their possible role in ibrutinib resistance. *BTK* and *PLCG2* mutations, were retrieved in 3/7 relapsed cases presenting a positive selection for *TP53* mutations at the relapse time (Supplementry Table S5). Overall, *BTK* and *PLCG2* mutations were discovered in 9/16 (56%) relapsed cases versus 3/22 (14%) patients under ibrutinib treatment (*P* = *0.006*, χ^2^ test; Supplementry Table S5).

In conclusion, here we provided evidence that only the co-presence of *TP53* deletion and mutations, the latter even with a low-VAF representation, and not the single aberrations have a negative prognostic impact in CLL patients under ibrutinib treatment. In practice, this finding points toward the need of a complete assessment of *TP53* aberrations to be performed in all CLL patients prior to start ibrutinib treatment. A lower threshold for reporting *TP53* mutations (e.g. VAF < 10%) must be evaluated in prospective clinical trial cohorts before it can be accepted as standard for routine practice. Moreover, low-VAF *TP53* mutations should be always confirmed by orthogonal assays (e.g. ddPCR) or by repetition [4].

## Supplementary information

Supplemental Material

## Data Availability

The data that support the findings of this study are available from the corresponding author upon request.
