# Supplementary material for: Clinical impact of TP53 disruption in chronic lymphocytic leukemia patients treated with ibrutinib: a campus CLL study
Source: Leukemia. 2023 Feb 18;37(4):914–8. doi: 10.1038/s41375-023-01845-9 (PMC10079531; doi:10.1038/s41375-023-01845-9)
Supplement: Supplementary file 1 — Supplemental Material [file 41375_2023_1845_MOESM1_ESM.pdf]

Supplemental Information

Clinical Impact of TP53 Disruption in Chronic Lymphocytic Leukemia Patients Treated with Ibrutinib. A Campus CLL Study

Riccardo Bomben[1], Francesca Maria Rossi[1], Filippo Vit[1], Tamara Bittolo[1], Antonella Zucchetto[1], Robel Papotti[2], Erika Tissino[1], Federico Pozzo[1], Massimo Degan[1], Jerry Polesel[3], Pietro Bulian[1], Roberto Marasca[4,5], Gianluigi Reda[6], Luca Laurenti[7], Jacopo Olivieri[8], Annalisa Chiarenza[9], Roberta Laureana[10], Massimiliano Postorino[10], Maria Ilaria Del Principe[10], Antonio Cuneo[11], Massimo Gentile[12,13], Fortunato Morabito[12,14], Gilberto Fronza[15], Agostino Tafuri[16], Francesco Zaja[17], Robin Foà[18], Francesco Di Raimondo[9], Giovanni Del Poeta[10], and Valter Gattei[1]

[1]Clinical and Experimental Onco-Haematology Unit, Centro di Riferimento Oncologico di Aviano (CRO) IRCCS, Italy;
[2]International PhD School in Clinical and Experimental Medicine, University of Modena and Reggio Emilia, Modena, Italy;
[3]Unit of Cancer Epidemiology, Centro di Riferimento Oncologico di Aviano (CRO) IRCCS, Aviano, Italy;
[4]Hematology Unit, Department of Oncology and Hematology, Azienda-Ospedaliero Universitaria (AOU) of Modena, Policlinico, Modena, Italy;
[5]Department of Medical and Surgical Sciences, University of Modena e Reggio Emilia;
[6]Division of Ematologia, Fondazione IRCCS Ca'Granda Ospedale Maggiore Policlinico di Milano, Milano, Italy;
[7]Fondazione Universitaria Policlinico A Gemelli di Roma, Roma, Italy;
[8]Clinica Ematologica, Centro Trapianti e Terapie Cellulari "Carlo Melzi" DISM, Azienda Ospedaliera Universitaria S. Maria Misericordia, Udine, Italy;
[9]Division of Hematology, Policlinico, Department of Surgery and Medical Specialties, University of Catania, Catania, Italy;
[10]Division of Haematology, University of Tor Vergata, Rome, Italy;
[11]Hematology Section, Department of Medical Sciences, University of Ferrara, Ferrara, Italy;
[12]Hematology Unit AO of Cosenza, Cosenza, Italy;
[13]Biothecnology Research Unit, AO of Cosenza, Cosenza, Italy
[14]Hematology Oncology Department, Augusta Victoria Hospital, East Jerusalem, Israel;
[15]Mutagenesis and Cancer Prevention Unit, IRCCS Ospedale Policlinico San Martino, Genoa, Italy;
[16]Department of Clinical and Molecular Medicine and Hematology, Sant'Andrea - University Hospital - Sapienza, University of Rome, Rome, Italy;
[17]Department of Medical, Surgical and Health Sciences, University of Trieste, Italy;
[18]Hematology, Department of Translational and Precision Medicine, 'Sapienza' University, Rome, Italy.

- **Supplemental Figures:**
  - **Figure S1. Clinical impact of sex, age, b2 microglobulin, hemoglobin, anemia, LDH, IGHV gene status, Rai stage, number of previous lines of therapy;**
  - **Figure S2. Variant specific ddPCR analysis of 3 different *TP53* mutations;**
  - **Figure S3. Clinical impact of *TP53* aberrations in ibrutinib-treated CLL.**

- **Supplemental Tables:**
  - **Table S1. Clinical features of the retrospective CLL cohort;**
  - **Table S2. Univariable and multivariable analyses of OS and PFS;**
  - **Table S3. *TP53* aberrations;**
  - **Table S4. NGS and ddPCR;**
  - **Table S5. Clonal evolution of *TP53* mutations in *TP53* mutated cases.**

TP53 mutations and bioinformatics pipeline of analysis

All the samples enrolled in this study were sequenced and analyzed with the same pipeline at the Clinical and Experimental Onco-Hematology Unit (Aviano, Italy).

For a more detailed description of the procedures for *TP53* mutational status determination, including functional evaluation and the applied bioinformatics pipeline, please refer to a recent publication of ours [1]. In addition, a custom script of the bioinformatics pipeline, as described herein and in [1]. is available at the website address https://github.com/gamabunta313/TP_SNP-calling/.

Briefly, analysis of *TP53* mutations was performed by next generation sequencing (NGS) with an amplicon-based strategy, covering exons 2-11, in keeping with the ERIC recommendations [2]. Specific primers were designed with the Primer3 program, and modified according to the Illumina (San Diego, CA) protocol [1]. Amplicon libraries were generated using a modified Illumina protocol starting from 40 ng of DNA (~6,000 diploid genomes), a quantity capable to successfully detect mutations below the 1% variant allele frequency (VAF) in the context of our procedures [1]. Multiplex PCR products were generated using Phusion High-Fidelity DNA Polymerase (Thermo Scientific, Milan, Italy) and subsequently tagged with specific index according to modified procedures for NexteraXT (DNALibrary Preparation kit, Illumina), as previously reported [3, 4]. Purified libraries were pooled, and paired-end sequenced in a MiSeq instrument (Illumina).

FASTQ files were aligned to the Hg19 reference with Burrows-Wheeler Aligner (BWA)-MEM algorithm, and allele variants were called by FreeBayes with non-stringent parameters [5–8]. A coverage ≥2,000X was obtained for each sample in 100% of the analyzed sequences. To calculate random/systematic errors, a database of 362 *TP53* wild-type (wt) cases was utilized [1]. As in Bomben et al [1], *TP53* mutations were accepted if both of the following conditions were fulfilled: i) with a VAF that outdistanced for at least 2.75 standard deviations the mean of the transformed VAF distribution related to any single nucleotide position of the *TP53* sequence; ii) validated by Fisher's exact test after Bonferroni correction ($P<0.01$). The minimal allelic fraction for *TP53* mutation calling was 0.3% [1].

The IARC TP53 Database (https://tp53.isb-cgc.org/) [9] was used to annotate *TP53* mutations, and to functionally evaluate *TP53* missense mutations for their capability to transcribe the *CDKN1A* gene [9, 10]. *TP53* mutated cases with less than 2% VAF were all confirmed by a second independent NGS run starting from DNA.

FISH analysis

Interphase FISH was performed on nuclei preparations of PB mononuclear cells, to detect 17p13.1 deletion (del17p) and 11q22.3 deletion (del11q) using the following locus specific probes, respectively: LSI-TP53 SpectrumOrange (17p13, 167 kb, *TP53* gene), LSI-ATM SpectrumGreen (11q22.3, 338 kb, ATM gene) (MetaSystems, Italy). Analyses were performed on a Eclipse 90i

Nikon fluorescent microscope equipped with a 100x planApo objective. In all cases, at least 200 interphase nuclei with well delineated fluorescent spots were examined and a threshold of ≥5% of nuclei (≥10% of nuclei for del 17p) was applied to discriminate between normal cases and cases bearing a specific chromosomal abnormality, in agreement with internal technical laboratory cut-offs, and as reported previously [11, 12].

Digital Droplet PCR

The detection of *TP53* mutations presented at very low frequencies was confirmed by Digital Droplet PCR (ddPCR) using specific Mutation Detection Assays according to manufacturer's instructions. A set of specific molecular probes were used to detect in the same assay the wild-type form of *TP53* and the specific mutations R248Q, R273H and Y234C (dHsaMDV2010127, dHsaMDV2010109, dHsaMDV2516900; Biorad), as born with low-VAF abundance in 22 CLL cases. The ddPCR experiments were performed on a QX200 system (BioRad). The reaction mixture was prepared in a final volume of 20 µL and comprised 10 µL of 2X ddPCR Super Mix for Probe (no dUTP), 1 µL of 20X target (FAM) and wild-type (HEX) primers/probe, 4 µL of water and 5 µL of template DNA with a concentration of 30 ng/µL. All samples, including negative controls (WT) were prepared in two replicates. After droplet generation with QX200 Droplet Generator, the PCR reaction was performed under the following conditions: 95 °C for 10 min; 40 cycles of 94 °C for 30 s and 57.5 °C for 60 s; 98 °C for 10 min, and in the end at 4°C (Veriti, Applied Biosystems). The droplets were read with QX200 Droplet Reader and QuantaSoft software was used to analyze the fractional abundance of each sample. The threshold was selected based on the WT controls, allowing a good separation between positive and negative events. Only fractional abundance percentage >0.1 and that outdistanced for at least 4 times respect to that calculated for WT samples were considered as truly mutated. ddPCR confirmed the presence of all the three *TP53* mutations investigated with similar fraction abundance (r=0.96; $P<0.0001$) in all the 22 CLL cases (Table S4 and Figure S2).

Other CLL characterizations

CLL patients were also characterized for gender, age, Rai staging, previous lines of therapy, anemia, β2-microglobulin (β2M), lactate-dehydrogenase (LDH) [3, 13–16]. Immunoglobulin-heavy-variable (IGHV) gene mutational status were analyzed by using the LymphoTrack IGHV Leader Somatic Hypermutation Assay MiSeq kit (Invivoscribe, San Diego, CA), according to the manufacturer's protocols [17]. Analysis of *BTK*, and *PLCG2* mutations was performed by NGS with an amplicon-based strategy, covering exons 11, 15, and 16, exons 12, 19, 20, 24, 27, and 30, respectively. Specific primers were designed with the Primer3 program, and modified according to the Illumina (San Diego, CA) protocol [1]. Amplicon libraries were generated using a modified Illumina protocol starting from 40 ng of DNA [1]. Multiplex PCR products were generated using Phusion High-Fidelity DNA Polymerase (Thermo Scientific, Milan, Italy) and subsequently tagged with specific index according to modified procedures for NexteraXT (DNALibrary Preparation kit, Illumina), as previously reported [3, 4]. Purified libraries were pooled, and paired-end sequenced in a

MiSeq instrument (Illumina). Figure S1 summarizes the clinical impact (OS) for all these variables in the cohort. In multivariable analyses, the variables “Rai staging” and “previous lines of therapy”, were dichotomized according to OS results, i.e. stage I-II vs III-IV and lines 0-1 vs >1, respectively.

Statistical analysis

All statistical analyses were performed by using standard methods [18–20]. OS was computed from date of ibrutinib start to death (events) or last follow-up (censoring). Progression-free survival (PFS) was calculated from the date of ibrutinib treatment initiation to progression (event) or last follow-up (censoring). The Cox proportional hazards regression models was chosen to assess the independent effect of covariates, treated as dichotomous, on OS or PFS, with stepwise procedure for selecting significant variables. Molecular studies were blinded to the study end points. Internal bootstrapping validations were as reported [18], by performing at least 500 replications.

References

1. Bomben R, Rossi FM, Vit F, Bittolo T, D'Agaro T, Zucchetto A, et al. TP53 mutations with low variant allele frequency predict short survival in Chronic Lymphocytic Leukemia. Clin Cancer Res. 2021;27:5566–76.

2. Malcikova J, Tausch E, Rossi D, Sutton LA, Soussi T, Zenz T, et al. ERIC recommendations for TP53 mutation analysis in chronic lymphocytic leukemia - Update on methodological approaches and results interpretation. Leukemia. 2018;32:1070–80.

3. D'Agaro T, Bittolo T, Bravin V, Dal Bo M, Pozzo F, Bulian P, et al. NOTCH1 mutational status in chronic lymphocytic leukaemia: clinical relevance of subclonal mutations and mutation types. Br J Haematol. 2018;182:597–602.

4. Pozzo F, Bittolo T, Vendramini E, Bomben R, Bulian P, Rossi FM, et al. NOTCH1-mutated chronic lymphocytic leukemia cells are characterized by a MYC-related overexpression of nucleophosmin 1 and ribosome-associated components. Leukemia. 2017;31:2407–15.

5. Garrison E, Marth G. Haplotype-based variant detection from short-read sequencing. arXiv. 2012;1207.

6. Bian X, Zhu B, Wang M, Hu Y, Chen Q, Nguyen C, et al. Comparing the performance of selected variant callers using synthetic data and genome segmentation. BMC Bioinformatics. 2018;19:429.

7. Liu F, Zhang Y, Zhang L, Li Z, Fang Q, Gao R, et al. Systematic comparative analysis of single-nucleotide variant detection methods from single-cell RNA sequencing data. Genome Biol. 2019;20:242.

8. Sandmann S, de Graaf AO, Karimi M, van der Reijden BA, Hellström-Lindberg E, Jansen JH, et al. Evaluating Variant Calling Tools for Non-Matched Next-Generation Sequencing Data. Sci Rep. 2017;7:43169.

9. Bouaoun L, Sonkin D, Ardin M, Hollstein M, Byrnes G, Zavadil J, et al. TP53 Variations in Human Cancers: New Lessons from the IARC TP53 Database and Genomics Data. Hum Mutat. 2016;37:865–76.

10. Leroy B, Fournier JL, Ishioka C, Monti P, Inga A, Fronza G, et al. The TP53 website: an integrative resource centre for the TP53 mutation database and TP53 mutant analysis. Nucleic Acids Res. 2013;41:D962–9.

11. Catovsky D, Richards S, Matutes E, Oscier D, Dyer M, Bezares RF, et al. Assessment of fludarabine plus cyclophosphamide for patients with chronic lymphocytic leukaemia (the LRF CLL4 Trial): a randomised controlled trial. Lancet (London, England). 2007;370:230–9.

12. Oscier D, Wade R, Davis Z, Morilla A, Best G, Richards S, et al. Prognostic factors identified three risk groups in the LRF CLL4 trial, independent of treatment allocation. Supplemental. Haematologica. 2010;95:1705–12.

13. Rossi FM, Zucchetto A, Tissino E, Dal Bo M, Bomben R, Caldana C, et al. CD49d expression identifies a chronic-lymphocytic leukemia subset with high levels of mobilized circulating CD34+ hemopoietic progenitors cells. Leukemia. 2014;28:705–8.

14. Gattei V, Bulian P, Del Principe MI, Zucchetto A, Maurillo L, Buccisano F, et al. Relevance of CD49d protein expression as overall survival and progressive disease prognosticator in chronic lymphocytic leukemia. Blood. 2008;111:865–73.

15. Dal Bo M, Bulian P, Bomben R, Zucchetto A, Rossi FM, Pozzo F, et al. CD49d prevails over the novel recurrent mutations as independent prognosticator of overall survival in chronic lymphocytic leukemia. Leukemia. 2016;30:2011–8.

16. Tissino E, Benedetti D, Herman SEM, ten Hacken E, Ahn IE, Chaffee KG, et al. Functional and clinical relevance of VLA-4 (CD49d/CD29) in ibrutinib-treated chronic lymphocytic leukemia. J Exp Med. 2018;215:681–97.

17. Leich E, Maier C, Bomben R, Vit F, Bosi A, Horn H, et al. Follicular lymphoma subgroups with and without t(14;18) differ in their N-glycosylation pattern and IGHV usage. Blood Adv. 2021;5:4890–900.

18. Efron B, Tibshirani R. Improvements on cross-validation: The .632+ bootstrap method. J Am Stat Assoc. 1997;92:548–60.

19. Van Houwelingen JC, Le Cessie S. Predictive value of statistical models. Stat Med. 1990;9:1303–25.

20. Ciampi A, Lawless JF, McKinney SM, Singhal K. Regression and recursive partition strategies in the analysis of medical survival data. J Clin Epidemiol. 1988;41:737–48.

Figure legends

Figure S1. Clinical impact of sex, age, b2 microglobulin, hemoglobin, anemia, LDH, IGHV gene status, Rai stage, number of previous lines of therapy. Kaplan-Meier curves comparing OS of patients from the ibrutinib treated cohort split in groups according to different prognostic markers. The number of patients in each group is reported; P values refer to the log-rank test.

Figure S2. Variant specific ddPCR analysis of 3 different *TP53* mutations. A) ddPCR for c.743G>A,p.R248Q. Fractional abundance percentage for 10 mutated cases and wild-type (WT) cases. B) ddPCR for c.701A>G,p.Y234C. Fractional abundance percentage for 3 mutated cases and WT cases. C) ddPCR for c.818G>A,p.R273H. Fractional abundance percentage for 9 mutated cases and WT cases. QuantaSoft software was used to calculate fractional abundance percentage. For WT cases fractional abundance percentage was calculated as the median value of 4 WT cases (R248Q), 4 WT cases (Y234C), and 7 WT cases (R273H). D) Correlation plot between variant allele frequency (VAF) % calculated by NGS and fractional abundance percentage calculated by ddPCR. P value refers to Rank correlation.

Figure S3. Clinical impact of *TP53* aberrations in ibrutinib-treated CLL. Kaplan-Meier curves comparing OS probabilities of 103 *TP53* wt (green line), 126 cases with *TP53* mutations irrespectively of VAF (red line). Cases with more than one mutation are classified according to the mutation with the highest VAF (see Table S3). The number of patients in each group is reported; P values refer to the log-rank test.

Figure S1

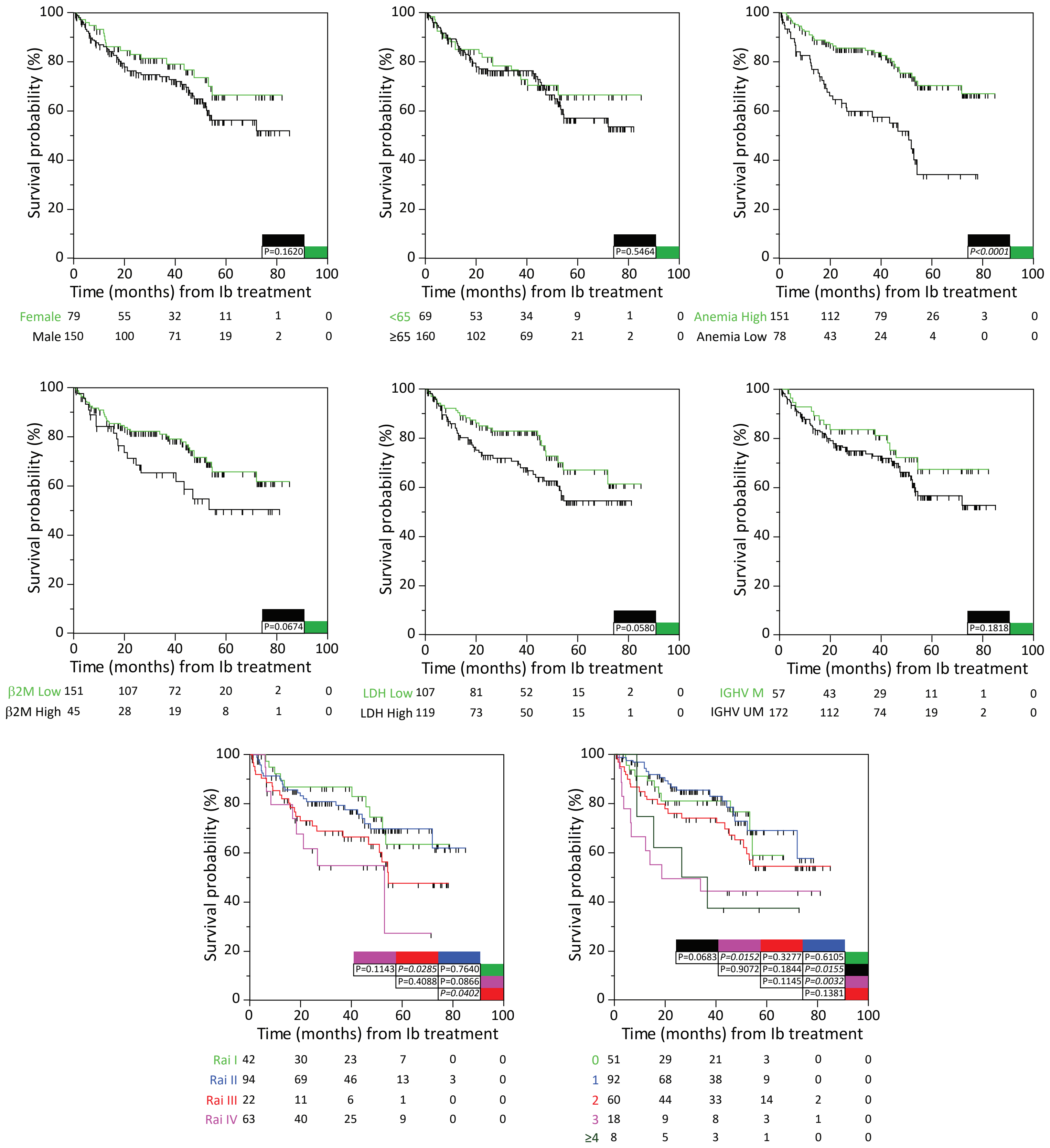

Figure S1. Clinical impact of sex, age, β2 microglobulin, hemoglobin, anemia, LDH, IGHV gene status, Rai stage, number of previous lines of therapy. Kaplan-Meier curves comparing OS of patients from the ibrutinib treated cohort split in groups according to different prognostic markers. The number of patients in each group is reported; P values refer to the log-rank test.

Figure S2

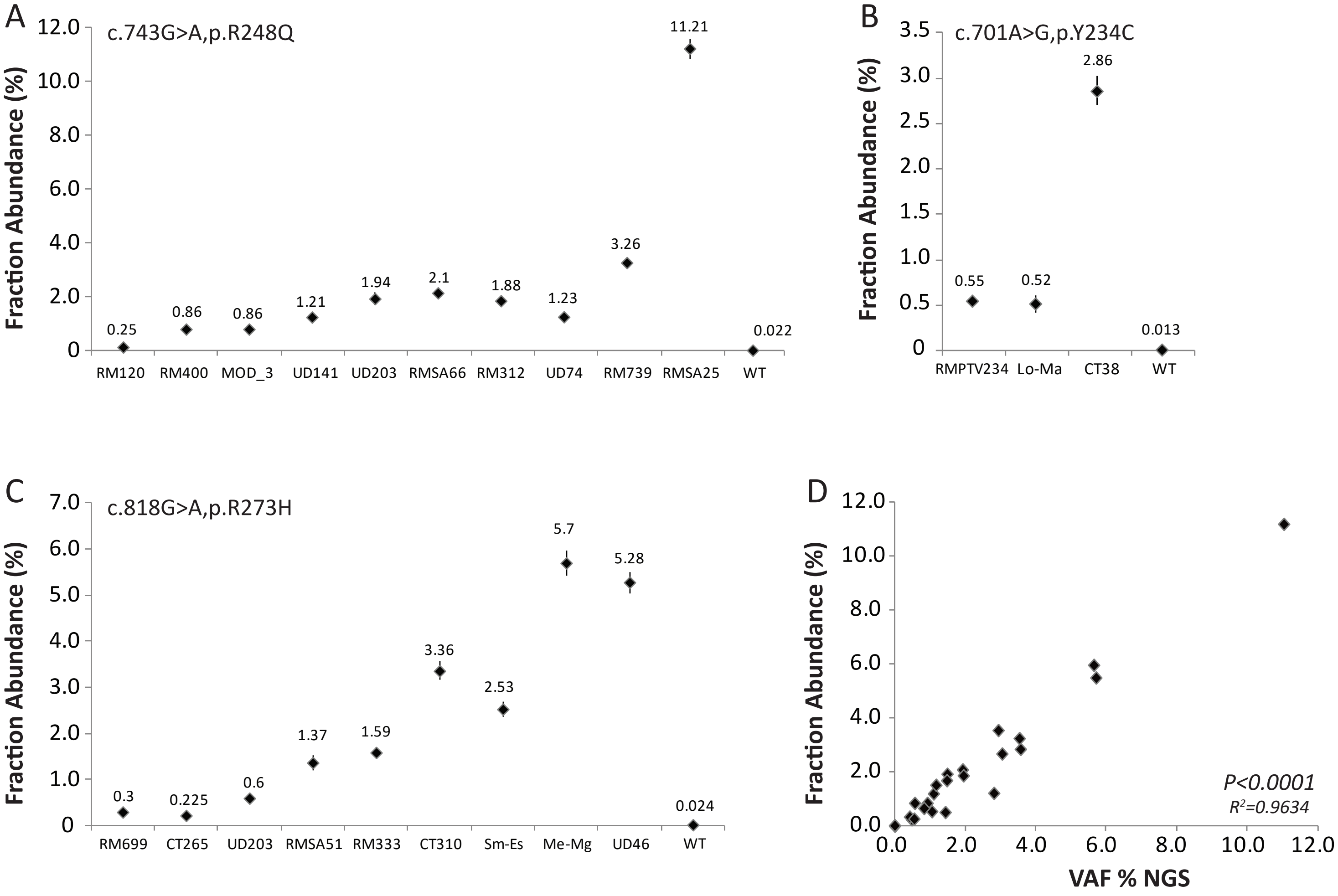

Figure S2. Variant specific ddPCR analysis of 3 different *TP53* mutations. A) ddPCR for c.743G>A,p.R248Q. Fractional abundance percentage for 10 mutated cases and wild-type (WT) cases. B) ddPCR for c.701A>G,p.Y234C. Fractional abundance percentage for 3 mutated cases and WT cases. C) ddPCR for c.818G>A,p.R273H. Fractional abundance percentage for 9 mutated cases and WT cases. QuantaSoft software was used to calculate fractional abundance percentage. For WT cases fractional abundance percentage was calculated as the median value of 4 WT cases (R248Q), 4 WT cases (Y234C), and 7 WT cases (R273H). D) Correlation plot between variant allele frequency (VAF) % calculated by NGS and fractional abundance percentage calculated by ddPCR. P value refers to Rank correlation.

Figure S3

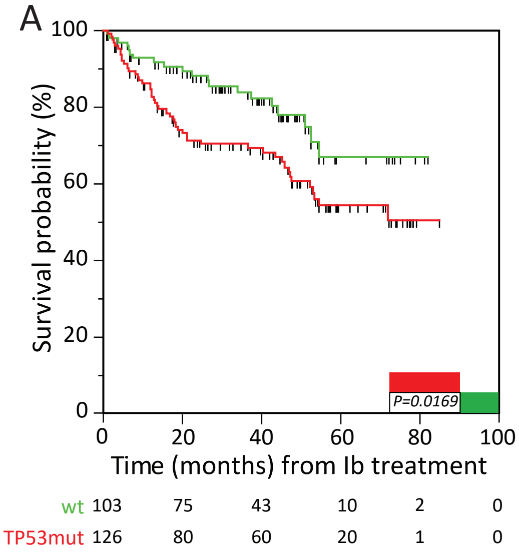

Figure S3. Clinical impact of *TP53* aberrations in ibrutinib-treated CLL. Kaplan-Meier curves comparing OS probabilities of 103 *TP53* wt (green line), 126 cases with *TP53* mutations irrespectively of VAF (red line). Cases with more than one mutation are classified according to the mutation with the highest VAF (see Table S3). The number of patients in each group is reported; P values refer to the log-rank test.

Table S1. Clinical features of the retrospective CLL cohort

		Total cohort n=229	
**Parameter**	**Category**	**N**	**%**
**Age**	<65	69	30.1
	$\geq$65	160	69.9
**Gender**	Female	79	34.5
	Male	150	65.5
**Previous Line of therapy**[a]	0	51	22.3
	1	92	40.2
	2	60	26.2
	3	18	7.9
	>3	8	3.5
**Rai stage**	0	0	0.0
	I	42	18.3
	II	94	41.0
	III	22	9.6
	IV	63	27.5
	Missing	8	3.5
TP53	Mutated high-VAF[b]	91	39.7
	Mutated low-VAF[b]	35	15.3
	wt	103	45.0
**del17p**	Present	74	32.3
	Absent	155	67.7
**del11q**	Present	56	24.5
	Absent	171	74.7
	Missing	2	0.9
**Hemoglobin g/L**	>120 for men / >110 for women	151	65.9
	$\leq$120 for men / $\leq$110 for women	78	34.1
**β2 microglobulin mg/L**	<5	151	65.9
	$\geq$5	45	19.7
	Missing	33	14.4
**Lactate dehydrogenase U/ml**	Normal	108	47.2
	Elevated	119	52.0
	Missing	2	0.9
**IGHV**	Mutated	76	33.2
	Unmutated	153	66.8

Abbreviations: IGHV unmutated, $\geq$98% identity with germ line;

[a] number of previous lines respect to ibrutinib treatment;

[b] high-VAF, $\geq$10.0% VAF; low-VAF, <10.0% VAF.

Table S2. Univariable and multivariable analyses of OS and PFS (n=219)

	UVA				MVA Model 1[a]				MVA Model 2[a]			
**OS**	**HR**	**LCI**	**UCI**	**P**	**HR**	**LCI**	**UCI**	**P**	**HR**	**LCI**	**UCI**	**P**
**Gender (Male)**	1.47	0.86	2.51	0.1644	-				-			
**Age (≥65 y)**	1.18	0.69	2.00	0.5489	-				-			
**Rai stage (I-II versus III-IV)**	1.82	1.12	2.94	***0.0147***	ni				ni			
**Previous Lines of therapy (0-1 versus >1)**	1.93	1.19	3.12	***0.0077***	1.92	1.17	3.14	***0.0093***	1.84	1.12	3.00	***0.0153***
**Anemia**	2.86	1.77	4.62	***<0.0001***	2.56	1.56	4.19	***0.0002***	2.54	1.50	4.16	***0.0002***
**β2 microglobulin (high)**	1.68	0.96	2.94	0.0706	-				-			
**LDH (high)**	1.61	0.98	2.65	0.0607	ni				ni	ni		
**IGHV (UM)**	1.49	0.83	2.68	0.1849	-				-	-		
**del11q (present)**	1.31	0.77	2.22	0.3233	-				-	-		
**del17p (present)**	1.67	1.04	2.70	***0.0355***	1.78	1.09	2.91	***0.0209***	not used			
***TP53* mutation (present)**	1.87	1.11	3.16	***0.0188***	not used				1.90	1.10	3.28	**0.0217**

Notes and abbreviations: Anemia, <110 g/L for women or <120 g/L for men; IGHV unmutated (UM), ≥98% identity with germ line; β2 microglobulin and LDH high, > upper normal level according to the different laboratories; OS, overall survival from ibrutinib start; PFS, progression free survival; UVA, univariable analysis; MVA, multivariable analysis; HR, Hazard Ratio; CI, confidence interval; LCI, 95% lower CI; UCI, 95% Upper CI; -: not used in the final model; ni: not included in the final model.

[a] Multivariable analysis was carried out using the following variables (n=219): Rai stage, previous lines of therapies, anemia, LDH, del17p (Model 1) or *TP53* mutations (Model 2)

	UVA				MVA Model 1[a]				MVA Model 2[a]			
**PFS**	**HR**	**LCI**	**UCI**	**P**	**HR**	**LCI**	**UCI**	**P**	**HR**	**LCI**	**UCI**	**P**
**Gender (Male)**	1.73	1.08	2.78	***0.0233***	1.78	1.09	2.89	***0.0207***	1.68	1.03	2.75	***0.0370***
**Age (≥65 y)**	1.03	0.66	1.61	0.8928	-				-			
**Rai stage (I-II versus III-IV)**	1.65	1.08	2.51	***0.0201***	ni				ni			
**Previous Lines of therapy (0-1 versus >1)**	2.34	1.54	3.55	***<0.0001***	2.41	1.57	3.7	***0.0001***	2.35	1.53	3.60	***0.0001***
**Anemia**	2.25	1.49	3.41	***0.0001***	2.18	1.43	3.35	***0.0003***	2.34	1.52	3.61	***0.0001***
**β2 microglobulin (high)**	1.92	1.06	3.47	***0.0308***	-				-			
**LDH (high)**	1.74	1.14	2.67	***0.0108***	ni				ni			
**IGHV (UM)**	1.81	1.07	3.08	***0.0272***	ni				1.94	1.10	3.42	***0.0231***
**del11q (present)**	1.21	0.76	1.93	0.4199	-				-			
**del17p (present)**	1.77	1.17	2.68	***0.0065***	1.82	1.19	2.78	***0.0057***	not used			
***TP53* mutation (present)**	1.59	1.03	2.48	***0.0385***	not used				ni			

Notes and abbreviations: Anemia, <110 g/L for women or <120 g/L for men; IGHV unmutated (UM), ≥98% identity with germ line; β2 microglobulin and LDH high, > upper normal level according to the different laboratories; OS, overall survival from ibrutinib start; PFS, progression free survival; UVA, univariable analysis; MVA, multivariable analysis; HR, Hazard Ratio; CI, confidence interval; LCI, 95% lower CI; UCI, 95% Upper CI; -: not used in the final model; ni: not included in the final model.

[a] Multivariable analysis was carried out using the following variables (n=219): gender, Rai stage, previous lines of therapies, anemia, LDH, IGHV, del17p (Model 1) or *TP53* mutations (Model 2)

Table S3. ***TP53*** **aberrations**

Sample_ID	TN/RR*	del17p	del17p %	Protein Change	Mutation Type	Variant allele frequency	high-VAF / low-VAF†	Coding‡
CT163	RR	1	47	?	splice	52.65	high-VAF	c.993+2T>C,p.?
CT163		1		R196*	nonsense	2.24	low-VAF	c.586C>T,p.R196*
CT163		1		H179R	missense	0.87	low-VAF	c.536A>G,p.H179R
CT168	RR	1	65	L252del	deletion	28.91	high-VAF	c.754_756del,p.L252del
CT168		1		R209Kfs*6	deletion	13.40	high-VAF	c.626_627del,p.R209Kfs*6
CT168		1		Y205*	deletion	2.34	low-VAF	c.615_618del,p.Y205*
CT168		1		R248W	missense	1.84	low-VAF	c.742C>T,p.R248W
CT168		1		R337H	missense	1.59	low-VAF	c.1010G>A,p.R337H
CT168		1		C277F	missense	0.63	low-VAF	c.830G>T,p.C277F
CT255	RR	1	48	R342*	nonsense	49.24	high-VAF	c.1024C>T,p.R342*
CT255		1		H193L	missense	8.00	low-VAF	c.578A>T,p.H193L
CT255		1		T125M	missense	1.82	low-VAF	c.374C>T,p.T125M
CT255		1		R337G	missense	0.40	low-VAF	c.1009C>G,p.R337G
CT265	RR	1	48	Y234C	missense	80.61	high-VAF	c.701A>G,p.Y234C
CT265		1		R248W	missense	0.83	low-VAF	c.742C>T,p.R248W
CT265		1		R273H	missense	0.55	low-VAF	c.818G>A,p.R273H
CT280	RR	1	83	R342*	nonsense	53.25	high-VAF	c.1024C>T,p.R342*
CT280		1		R248Q	missense	12.57	high-VAF	c.743G>A,p.R248Q
CT291	RR	0	0	D7H	missense	1.32	low-VAF	c.19G>C,p.D7H
CT304	RR	1	30	R209Kfs*6	deletion	74.65	high-VAF	c.626_627del,p.R209Kfs*6
CT304		1		D281E	missense	15.14	high-VAF	c.843C>A,p.D281E
CT310	RR	0	0	R273H	missense	2.94	low-VAF	c.818G>A,p.R273H
CT338	RR	0	3	R158_Y163delinsN	complex	8.06	low-VAF	c.472_487delinsA,p.R158_Y163delinsN
CT338		0		I255F	missense	4.23	low-VAF	c.763A>T,p.I255F
CT338		0		R248W	missense	0.49	low-VAF	c.742C>T,p.R248W
CT366	RR	1	82	P72Wfs*50	deletion	7.09	low-VAF	c.214_217del,p.P72Wfs*50
CT38	RR	1	70	S183*	nonsense	19.90	high-VAF	c.548C>G,p.S183*
CT38		1		?	splice	7.70	low-VAF	c.560-2A>T,p.?
CT38		1		Y163C	missense	6.73	low-VAF	c.488A>G,p.Y163C
CT38		1		Y234S	missense	6.56	low-VAF	c.701A>C,p.Y234S
CT38		1		?	splice	3.62	low-VAF	c.375+1G>T,p.?
CT38		1		Y234C	missense	3.57	low-VAF	c.701A>G,p.Y234C
CT38		1		V157_R158del	deletion	2.67	low-VAF	c.470_475del,p.V157_R158del
CT387	RR	1	51	A159V	missense	42.96	high-VAF	c.476C>T,p.A159V
CT387		1		C277F	missense	9.91	low-VAF	c.830G>T,p.C277F
CT387		1		M237V	missense	2.01	low-VAF	c.709A>G,p.M237V
CT387		1		T253N	missense	1.03	low-VAF	c.758C>A,p.T253N
CT387		1		K120N	missense	0.79	low-VAF	c.360G>T,p.K120N
CT398	RR	0	4	?	splice	23.95	high-VAF	c.672+1G>A,p.?
CT399	RR	0	0	R273L	missense	0.51	low-VAF	c.818G>T,p.R273L
CT403	TN	0	9	R175H	missense	28.17	high-VAF	c.524G>A,p.R175H
CT407	RR	1	95	?	splice	90.10	high-VAF	c.673-2A>G,p.?
CT413	RR	1	98	?	splice	99.61	high-VAF	c.375+1G>T,p.?
CT431	TN	1	17	C176Y	missense	4.49	low-VAF	c.527G>A,p.C176Y
CT436	TN	0	0	R306*	nonsense	46.19	high-VAF	c.916C>T,p.R306*
CT436		0		Q167Pfs*13	deletion	39.33	high-VAF	c.500_501del,p.Q167Pfs*13
CT440	TN	1	74	Q331*	nonsense	97.70	high-VAF	c.991C>T,p.Q331*
CT453	RR	0	0	G245S	missense	1.07	low-VAF	c.733G>A,p.G245S
CT455	RR	0	0	Y205D	missense	4.52	low-VAF	c.613T>G,p.Y205D
CT60	RR	1	26	G245S	missense	35.76	high-VAF	c.733G>A,p.G245S
CT60		1		G245V	missense	4.32	low-VAF	c.734G>T,p.G245V
CT60		1		K120Nfs*3	deletion	4.25	low-VAF	c.360del,p.K120Nfs*3
CT60		1		R283P	missense	2.79	low-VAF	c.848G>C,p.R283P
CT60		1		H214R	missense	2.75	low-VAF	c.641A>G,p.H214R
CT60		1		?	splice	1.67	low-VAF	c.673-2A>T,p.?
CT92	RR	0	2	G244D	missense	10.40	high-VAF	c.731G>A,p.G244D
Gh-Di	RR	0	0	Y234C	missense	15.61	high-VAF	c.701A>G,p.Y234C
Li-Giu	RR	0	0	L188Rfs*59	deletion	24.64	high-VAF	c.563del,p.L188Rfs*59
Li-Giu		0		E286G	missense	1.51	low-VAF	c.857A>G,p.E286G
Lo-Ma	RR	0	4	R248W	missense	1.62	low-VAF	c.742C>T,p.R248W
Lo-Ma		0		Y234C	missense	1.44	low-VAF	c.701A>G,p.Y234C
Me-Mg	RR	0	0	A276_C277del	deletion	23.36	high-VAF	c.826_831del,p.A276_C277del
Me-Mg		0		N239Vfs*9	insertion	8.69	low-VAF	c.713_714dup,p.N239Vfs*9
Me-Mg		0		R273H	missense	5.65	low-VAF	c.818G>A,p.R273H
Me-Mg		0		P152R	missense	0.55	low-VAF	c.455C>G,p.P152R
MODENA_16§	TN	0	0	?	splice	94.82	high-VAF	c.375+2T>G,p.?
MODENA_2	TN	0	6	R248Q	missense	44.98	high-VAF	c.743G>A,p.R248Q
MODENA_3	RR	1	68	?	splice	10.75	high-VAF	c.673-2A>C,p.?

MODENA_3		1		Y234C	missense	8.66	low-VAF	c.701A>G,p.Y234C
MODENA_3		1		?	splice	7.17	low-VAF	c.673-2A>T,p.?
MODENA_3		1		M237I	missense	2.18	low-VAF	c.711G>A,p.M237I
MODENA_3		1		K132T	missense	2.02	low-VAF	c.395A>C,p.K132T
MODENA_3		1		L188Kfs*49	deletion	1.68	low-VAF	c.560_590del,p.L188Kfs*49
MODENA_3		1		M237I	missense	1.67	low-VAF	c.711G>T,p.M237I
MODENA_3		1		G244D	missense	1.60	low-VAF	c.731G>A,p.G244D
MODENA_3		1		?	splice	1.52	low-VAF	c.672+1G>T,p.?
MODENA_3		1		Y234S	missense	1.45	low-VAF	c.701A>C,p.Y234S
MODENA_3		1		Q144*	nonsense	1.41	low-VAF	c.430C>T,p.Q144*
MODENA_3		1		S261Mfs*84	deletion	1.04	low-VAF	c.782del,S261Mfs*84
MODENA_3		1		?	splice	0.99	low-VAF	c.673-2A>G,p.?
MODENA_3		1		R248Q	missense	0.93	low-VAF	c.743G>A,p.R248Q
MODENA_3		1		G244R	missense	0.86	low-VAF	c.730G>C,p.G244R
MODENA_3		1		E51*	nonsense	0.71	low-VAF	c.151G>T,p.E51*
MODENA_3		1		?	splice	0.34	low-VAF	c.560-2A>C,p.?
MODENA_3		1		N239T	missense	0.31	low-VAF	c.716A>C,p.N239T
MODENA_4	RR	1	85	M237I	missense	86.74	high-VAF	c.711G>T,p.M237I
MODENA_7	RR	1	26	A276P	missense	42.82	high-VAF	c.826G>C,p.A276P
MODENA_7		1		C176W	missense	1.09	low-VAF	c.528C>G,p.C176W
MODENA_9	RR	1	48	P152Rfs*18	deletion	55.32	high-VAF	c.455delC,p.P152Rfs*18
RM113§	RR	0	6	V272L	missense	81.34	high-VAF	c.814G>T,p.V272L
RM120	RR	0	0	G245D	missense	2.58	low-VAF	c.734G>A,p.G245D
RM120		0		R248Q	missense	0.48	low-VAF	c.743G>A,p.R248Q
RM13	RR	0	5	R306*	nonsense	2.06	low-VAF	c.916C>T,p.R306*
RM152	RR	1	82	D281V	missense	65.84	high-VAF	c.842A>T,p.D281V
RM152		1		R248W	missense	5.44	low-VAF	c.742C>T,p.R248W
RM152		1		H214R	missense	1.28	low-VAF	c.641A>G,p.H214R
RM28	RR	0	0	R175H	missense	26.14	high-VAF	c.524G>A,p.R175H
RM28		0		N239S	missense	25.41	high-VAF	c.716A>G,p.N239S
RM295	RR	0	0	R282W	missense	7.31	low-VAF	c.844C>T,p.R282W
RM309	RR	0	0	P58Sfs*5	insertion	1.61	low-VAF	c.171_172insT,p.P58Sfs*5
RM312	RR	1	16	V157G	missense	11.71	high-VAF	c.470T>G,p.V157G
RM312		1		R248Q	missense	1.95	low-VAF	c.743G>A,p.R248Q
RM333	RR	0	0	H178D	missense	14.81	high-VAF	c.532C>G,p.H178D
RM333		0		I255T	missense	10.41	high-VAF	c.764T>C,p.I255T
RM333		0		V216L	missense	9.77	low-VAF	c.646G>T,p.V216L
RM333		0		Y234C	missense	8.05	low-VAF	c.701A>G,p.Y234C
RM333		0		?	splice	3.96	low-VAF	c.673-2A>G,p.?
RM333		0		K319*	nonsense	2.48	low-VAF	c.955A>T,p.K319*
RM333		0		K132M	missense	1.82	low-VAF	c.395A>T,p.K132M
RM333		0		?	splice	1.81	low-VAF	c.673-2A>T,p.?
RM333		0		?	splice	1.71	low-VAF	c.673-2A>C,p.?
RM333		0		R273H	missense	1.48	low-VAF	c.818G>A,p.R273H
RM333		0		K132R	missense	1.29	low-VAF	c.395A>G,p.K132R
RM333		0		F270I	missense	1.16	low-VAF	c.808T>A,p.F270I
RM333		0		D281V	missense	1.06	low-VAF	c.842A>T,p.D281V
RM333		0		N131I	missense	0.88	low-VAF	c.392A>T,p.N131I
RM333		0		V272M	missense	0.84	low-VAF	c.814G>A,p.V272M
RM333		0		?	splice	0.78	low-VAF	c.560-2A>T,p.?
RM333		0		?	splice	0.72	low-VAF	c.783-2A>C,p.?
RM333		0		Q192*	nonsense	0.67	low-VAF	c.574C>T,p.Q192*
RM333		0		C277F	missense	0.42	low-VAF	c.830G>T,p.C277F
RM394	RR	0	0	Y163C	missense	3.35	low-VAF	c.488A>G,p.Y163C
RM400	RR	0	4	R248G	missense	12.27	high-VAF	c.742C>G,p.R248G
RM400		0		D281Y	missense	8.20	low-VAF	c.841G>T,p.D281Y
RM400		0		M246V	missense	1.77	low-VAF	c.736A>G,p.M246V
RM400		0		R248W	missense	0.83	low-VAF	c.742C>T,p.R248W
RM400		0		H179L	missense	0.73	low-VAF	c.536A>T,p.H179L
RM400		0		R248Q	missense	0.57	low-VAF	c.743G>A,p.R248Q
RM400		0		Y236N	missense	0.30	low-VAF	c.706T>A,p.Y236N
RM459	RR	0	5	I195T	missense	38.46	high-VAF	c.584T>C,p.I195T
RM576	RR	1	13	S149fs*19	deletion	11.11	high-VAF	c.445_451del,p.S149fs*19
RM576		1		W53*	nonsense	5.10	low-VAF	c.158G>A,p.W53*
RM629	RR	1	50	R280G	missense	88.68	high-VAF	c.838A>G,p.R280G
RM653	RR	1	68	G245S	missense	94.52	high-VAF	c.733G>A,p.G245S
RM655	RR	0	5	C238Y	missense	1.15	low-VAF	c.713G>A,p.C238Y
RM664	RR	1	53	?	splice	47.46	high-VAF	c.673-1G>A,p.?
RM664		1		F109S	missense	37.70	high-VAF	c.326T>C,p.F109S
RM664		1		?	splice	1.05	low-VAF	c.375G>A,p.T125T
RM684	RR	1	88	H179R	missense	11.11	high-VAF	c.536A>G,p.H179R
RM699	RR	1	23	R196Q	missense	43.62	high-VAF	c.587G>A,p.R196Q
RM699		1		S241Y	missense	4.62	low-VAF	c.722C>A,p.S241Y

RM699		1		P278T	missense	1.69	low-VAF	c.832C>A,p.P278T
RM699		1		I195T	missense	0.89	low-VAF	c.584T>C,p.I195T
RM699		1		R248W	missense	0.88	low-VAF	c.742C>T,p.R248W
RM699		1		C275R	missense	0.59	low-VAF	c.823T>C,p.C275R
RM699		1		R273H	missense	0.42	low-VAF	c.818G>A,p.R273H
RM739	RR	0	8	R273C	missense	38.47	high-VAF	c.817C>T,p.R273C
RM739		0		R248Q	missense	3.54	low-VAF	c.743G>A,p.R248Q
RM739		0		C176Y	missense	0.59	low-VAF	c.527G>A,p.C176Y
RM739		0		T253P	missense	0.57	low-VAF	c.757A>C,p.T253P
RM739		0		A276P	missense	0.50	low-VAF	c.826G>C,p.A276P
RM76	RR	1	94	V173M	missense	88.89	high-VAF	c.517G>A,p.V173M
RM767	TN	1	69	R175H	missense	75.03	high-VAF	c.524G>A,p.R175H
RM770	RR	1	85	?	splice	72.22	high-VAF	c.920-2A>G,p.?
RM770		1		S241C	missense	2.89	low-VAF	c.722C>G,p.S241C
RM770		1		?	splice	2.15	low-VAF	c.376-2A>G,p.?
RM781[§]	RR	0	0	Y205H	missense	86.26	high-VAF	c.613T>C,p.Y205H
RMGEM118	TN	1	20.5	S90Lfs*59	insertion	85.61	high-VAF	c.267dup,p.S90Lfs*59
RMGEM128	TN	0	0.5	I195N	missense	65.83	high-VAF	c.584T>A,p.I195N
RMGEM142	TN	1	20.5	W91*	nonsense	84.70	high-VAF	c.272G>A,p.W91*
RMGEM162	RR	1	34	L344R	missense	15.35	high-VAF	c.1031T>G,p.L344R
RMGEM162		1		D228*	insertion	14.15	high-VAF	c.681dup,p.D228*
RMGEM162		1		G245S	missense	9.96	low-VAF	c.733G>A,p.G245S
RMGEM162		1		K132R	missense	0.90	low-VAF	c.395A>G,p.K132R
RMGEM18	RR	1	88	C176F	missense	80.44	high-VAF	c.527G>T,p.C176F
RMGEM36	TN	0	0	R209Kfs*6	deletion	12.28	high-VAF	c.626_627del,p.R209Kfs*6
RMGEM38	RR	0	9	G245D	missense	3.42	low-VAF	c.734G>A,p.G245D
RMGEM49	TN	1	93	V218E	missense	80.61	high-VAF	c.653T>A,p.V218E
RMGEM54	RR	0	0.5	V218delinsDL	insertion	3.43	low-VAF	c.652_653insACC,p.V218delinsDL
RMGEM6	RR	0	0	P250L	missense	1.42	low-VAF	c.749C>T,p.P250L
RMPTV140	RR	1	62	R273C	missense	88.79	high-VAF	c.817C>T,p.R273C
RMPTV144	RR	1	85	N235_N239del	deletion	97.04	high-VAF	c.704_718del,p.N235_N239del
RMPTV164	TN	1	94	R306*	nonsense	78.40	high-VAF	c.916C>T,p.R306*
RMPTV234	RR	1	16	V173L	missense	4.22	low-VAF	c.517G>T,p.V173L
RMPTV234		1		V173G	missense	1.22	low-VAF	c.518T>G,p.V173G
RMPTV234		1		Y234C	missense	1.05	low-VAF	c.701A>G,p.Y234C
RMPTV234		1		R196L	complex	0.89	low-VAF	c.587_588delinsTT,p.R196L
RMPTV234		1		G244A	missense	0.53	low-VAF	c.731G>C,p.G244A
RMPTV241	TN	0	8	R280T	missense	38.78	high-VAF	c.839G>C,p.R280T
RMPTV261	RR	1	15	Q331Rfs*14	deletion	2.38	low-VAF	c.991del,p.Q331Rfs*14
RMPTV261		1		E286K	missense	2.28	low-VAF	c.856G>A,p.E286K
RMPTV261		1		N131del	deletion	2.19	low-VAF	c.393_395del,p.N131del
RMPTV277	RR	1	40	P250S	complex	12.22	high-VAF	c.747_748delinsTT,p.R249_P250delinsSS
RMPTV277		1		G245S	missense	8.71	low-VAF	c.733G>A,p.G245S
RMPTV277		1		Q165Hfs*5	deletion	3.25	low-VAF	c.495del,p.Q165Hfs*5
RMPTV277		1		L145Rfs*25	deletion	2.34	low-VAF	c.434del,p.L145Rfs*25
RMPTV277		1		?	splice	1.53	low-VAF	c.782+1G>T,p.?
RMPTV277		1		E286Q	missense	0.68	low-VAF	c.856G>C,p.E286Q
RMPTV277		1		R273C	missense	0.65	low-VAF	c.817C>T,p.R273C
RMPTV277		1		Y234*	nonsense	0.49	low-VAF	c.702C>G,p.Y234*
RMPTV293	TN	1	89	L32Rfs*11	insertion	86.79	high-VAF	c.94_95insG,p.L32Rfs*11
RMPTV312	TN	1	80	H179D	missense	53.34	high-VAF	c.535C>G,p.H179D
RMPTV312		1		S241F	missense	43.60	high-VAF	c.722C>T,p.S241F
RMPTV322	RR	0	0	I255N	missense	1.69	low-VAF	c.764T>A,p.I255N
RMPTV324	TN	0	7	R175C	missense	49.15	high-VAF	c.523C>T,p.R175C
RMPTV341	RR	1	93	H193R	missense	91.05	high-VAF	c.578A>G,p.H193R
RMPTV341		1		Y220C	missense	1.47	low-VAF	c.659A>G,p.Y220C
RMPTV353	TN	0	0	I232T	missense	39.17	high-VAF	c.695T>C,p.I232T
RMPTV353		0		I195F	missense	4.62	low-VAF	c.583A>T,p.I195F
RMPTV354	RR	1	84	R280*	nonsense	90.11	high-VAF	c.838A>T,p.R280*
RMPTV354		1		N239*	insertion	5.86	low-VAF	c.714dup,p.N239*
RMPTV366	TN	1	95	E224*	insertion	68.64	high-VAF	c.669dup,p.E224*
RMPTV366		1		S96Yfs*53	insertion	13.01	high-VAF	c.286_287insA,p.S96Yfs*53
RMPTV366		1		R282W	missense	2.40	low-VAF	c.844C>T,p.R282W
RMPTV42	RR	0	0	V157A	missense	6.31	low-VAF	c.470T>C,p.V157A
RMPTV42		0		E294*	nonsense	1.31	low-VAF	c.880G>T,p.E294*
RMPTV441	TN	1	47	R282W	missense	30.70	high-VAF	c.844C>T,p.R282W
RMPTV472	TN	1	96	Q331Rfs*14	deletion	84.23	high-VAF	c.990del,p.Q331Rfs*14
RMPTV472		1		Y327*	nonsense	84.08	high-VAF	c.981T>G,p.Y327*
RMPTV51	RR	1	82	T102Pfs*21	deletion	11.91	high-VAF	c.304del,p.T102Pfs*21
RMPTV53	TN	1	22	?	splice	63.10	high-VAF	c.560-1G>A,p.?
RMPTV8	RR	0	0	R249T	missense	0.56	low-VAF	c.746G>C,p.R249T
RMSA18	RR	0	0	S127F	missense	13.04	high-VAF	c.380C>T,p.S127F
RMSA18		0		?	splice	8.86	low-VAF	c.560-2A>T,p.?

RMSA18		0		Y220C	missense	1.55	low-VAF	c.659A>G,p.Y220C
RMSA18		0		C238G	missense	0.59	low-VAF	c.712T>G,p.C238G
RMSA25	RR	0	3	R248Q	missense	11.04	high-VAF	c.743G>A,p.R248Q
RMSA30	RR	1	83	T230Yfs*10	insertion	57.61	high-VAF	c.687dup,p.T230Yfs*10
RMSA30		1		K320*	nonsense	4.93	low-VAF	c.958A>T,p.K320*
RMSA30		1		P152L	missense	2.91	low-VAF	c.455C>T,p.P152L
RMSA30		1		G245D	missense	1.65	low-VAF	c.734G>A,p.G245D
RMSA30		1		V272M	missense	1.63	low-VAF	c.814G>A,p.V272M
RMSA30		1		P278R	missense	0.53	low-VAF	c.833C>G,p.P278R
RMSA30		1		T18Hfs*26	deletion	0.40	low-VAF	c.52del,T18Hfs*26
RMSA41	TN	0	3	S240G	missense	43.34	high-VAF	c.718A>G,p.S240G
RMSA45	TN	0	0	T125A	missense	7.20	low-VAF	c.373A>G,p.T125A
RMSA45		0		C242F	missense	1.79	low-VAF	c.725G>T,p.C242F
RMSA45		0		F270S	missense	1.64	low-VAF	c.809T>C,p.F270S
RMSA48	RR	1	80	I195T	missense	63.82	high-VAF	c.584T>C,p.I195T
RMSA48		1		V274L	missense	7.88	low-VAF	c.820G>C,p.V274L
RMSA51	RR	0	7	E286D	missense	31.07	high-VAF	c.858A>C,p.E286D
RMSA51		0		I255N	missense	25.08	high-VAF	c.764T>A,p.I255N
RMSA51		0		R273C	missense	8.05	low-VAF	c.817C>T,p.R273C
RMSA51		0		S183*	nonsense	1.23	low-VAF	c.548C>G,p.S183*
RMSA51		0		R273H	missense	1.17	low-VAF	c.818G>A,p.R273H
RMSA51		0		R248Q	missense	0.70	low-VAF	c.743G>A,p.R248Q
RMSA51		0		C135W	missense	0.39	low-VAF	c.405C>G,p.C135W
RMSA53	TN	1	83	C176W	missense	92.16	high-VAF	c.528C>G,p.C176W
RMSA54	TN	0	0	G245S	missense	1.43	low-VAF	c.733G>A,p.G245S
RMSA66	RR	0	4	R248Q	missense	1.93	low-VAF	c.743G>A,p.R248Q
RMSA67	TN	1	96	R333H	missense	95.70	high-VAF	c.998G>A,p.R333H
RMSA67		1		T284P	missense	89.19	high-VAF	c.850A>C,p.T284P
Sm-Es	RR	0	0	G244S	missense	46.58	high-VAF	c.730G>A,p.G244S
Sm-Es		0		R273H	missense	3.04	low-VAF	c.818G>A,p.R273H
TS134	TN	0	0	?	splice	15.17	high-VAF	c.560-1G>A,p.?
TS208	RR	1	11	Q167Hfs*12	deletion	5.59	low-VAF	c.501_505del,p.Q167Hfs*12
TS242	TN	1	70	E271K	missense	54.66	high-VAF	c.811G>A,p.E271K
TS280	TN	1	12	G199Efs*48	deletion	65.00	high-VAF	c.594del,p.G199Efs*48
TS290	RR	0	0	C277F	missense	1.19	low-VAF	c.830G>T,p.C277F
TS33	RR	1	30	G245S	missense	13.12	high-VAF	c.733G>A,p.G245S
TS368	RR	0	3	?	complex	40.84	high-VAF	994-2del,p.?
TS368		0		L257P	missense	26.05	high-VAF	c.770T>C,p.L257P
TS88	RR	0	0	R213*	nonsense	0.99	low-VAF	c.637C>T,p.R213*
TS88		0		V274F	missense	0.97	low-VAF	c.820G>T,p.V274F
UD101	TN	1	98	C242F	missense	75.71	high-VAF	c.725G>T,p.C242F
UD115	RR	1	97	N247I	missense	91.77	high-VAF	c.740A>T,p.N247I
UD126	TN	1	25	R282W	missense	6.15	low-VAF	c.844C>T,p.R282W
UD13[§]	RR	0	0	W146del	deletion	82.00	high-VAF	c.436_438del,p.W146del
UD141	RR	0	4	S149Tfs*30	deletion	12.60	high-VAF	c.444_448del,p.S149Tfs*30
UD141		0		R248W	missense	1.31	low-VAF	c.742C>T,p.R248W
UD141		0		R248Q	missense	1.10	low-VAF	c.743G>A,p.R248Q
UD141		0		C176Y	missense	0.69	low-VAF	c.527G>A,p.C176Y
UD15	RR	1	48	C238S	missense	10.89	high-VAF	c.712T>A,p.C238S
UD15		1		R248W	missense	9.52	low-VAF	c.742C>T,p.R248W
UD15		1		R196*	nonsense	2.35	low-VAF	c.586C>T,p.R196*
UD15		1		V157F	missense	0.78	low-VAF	c.469G>T,p.V157F
UD15		1		L194R	missense	0.59	low-VAF	c.581T>G,p.L194R
UD150	RR	0	9	I195N	missense	1.85	low-VAF	c.584T>A,p.I195N
UD161	TN	1	95	I232T	missense	82.22	high-VAF	c.695T>C,p.I232T
UD18	RR	0	0	R248W	missense	6.55	low-VAF	c.742C>T,p.R248W
UD18		0		K132M	missense	2.03	low-VAF	c.395A>T,p.K132M
UD18		0		R273C	missense	0.59	low-VAF	c.817C>T,p.R273C
UD185	RR	1	86	N247I	missense	96.79	high-VAF	c.740A>T,p.N247I
UD185		1		E51Nfs*72	deletion	2.09	low-VAF	c.151del,p.E51Nfs*72
UD203	RR	1	17	Y234C	missense	38.86	high-VAF	c.701A>G,p.Y234C
UD203		1		E286Kfs*59	deletion	4.06	low-VAF	c.856del,p.E286Kfs*59
UD203		1		R248Q	missense	1.49	low-VAF	c.743G>A,p.R248Q
UD203		1		R273H	missense	0.83	low-VAF	c.818G>A,p.R273H
UD216	RR	0	8	R248Q	missense	41.25	high-VAF	c.743G>A,p.R248Q
UD216		0		Q52Pfs*71	complex	40.21	high-VAF	c.155_158delinsCAT,p.Q52Pfs*71
UD30	RR	0	0	D281V	missense	2.50	low-VAF	c.842A>T,p.D281V
UD30		0		Y205C	missense	1.22	low-VAF	c.614A>G,p.Y205C
UD30		0		C135*	complex	0.93	low-VAF	c.404_406del,p.C135*
UD46	RR	1	78	R273H	missense	5.71	low-VAF	c.818G>A,p.R273H
UD46		1		R213*	nonsense	1.75	low-VAF	c.637C>T,p.R213*
UD74		0		R273C	missense	29.53	high-VAF	c.817C>T,p.R273C
UD74		0		R248Q	missense	2.81	low-VAF	c.743G>A,p.R248Q

UD74		0		R249S	missense	0.53	low-VAF	c.747G>C,p.R249S
UD74		0		C277F	missense	0.59	low-VAF	c.830G>T,p.C277F
UD74	RR	0	4	V157F	missense	0.42	low-VAF	c.469G>T,p.V157F
UD76	RR	1	65	R248G	missense	73.26	high-VAF	c.742C>G,p.R248G
UD85	RR	0	6	R175H	missense	1.53	low-VAF	c.524G>A,p.R175H
UD85		0		C176Y	missense	0.88	low-VAF	c.527G>A,p.C176Y
UD85		0		C275G	missense	0.67	low-VAF	c.823T>G,p.C275G
UD85		0		C277F	missense	0.52	low-VAF	c.830G>T,p.C277F
UD85		0		D259Y	missense	0.35	low-VAF	c.775G>T,p.D259Y
CT151	RR	1	72					wt
CT447	TN	1	18					wt
MODENA_15	RR	1	88					wt
RM422	RR	1	88					wt
RMPTV133	RR	1	78					wt
RMPTV199	RR	1	24					wt
TS235	RR	1	64					wt
TS31	RR	1	11					wt

* TN, Treatment naive; RR, Relapsed Refractory;

† high-VAF, $\geq$10.0% VAF; low-VAF, <10.0% VAF;

‡ According to Human Genome Variation Society (HGVS) nomenclature. https://www.mutalyzer.nl/;

§ Cases with very high VAF percentage (>81%) in absence of concomitant del17p indicative of duplication of the mutated allele i.e. copy neutral loss of heterozygosity; copy neutral loss of heterozygosity was also consistent with what observed for the P72R polymorphysm: in all 4 cases the VAF of one of the

For cases with multiple mutations the mutation with the highest VAF is highlighted (grey cell fill color).

Table S4. NGS and ddPCR

Sample_ID	Protein Change	NGS Variant allele frequency	ddPCR Fraction Abundance (%)	ddPCR Positive events
RM120	R248Q	0.48	0.25	213
RM400	R248Q	0.57	0.86	312
MODENA_3	R248Q	0.93	0.86	276
UD141	R248Q	1.10	1.21	610
UD203	R248Q	1.49	1.94	923
RMSA66	R248Q	1.93	2.1	1840
RM312	R248Q	1.95	1.88	662
UD74	R248Q	2.81	1.23	529
RM739	R248Q	3.54	3.26	1170
RMSA25	R248Q	11.04	11.21	3727
WT_R248Q*	R248Q	0.00	0.022	7.75
RMPTV234	Y234C	1.05	0.55	267
Lo-Ma	Y234C	1.44	0.52	353
CT38	Y234C	3.57	2.86	1203
WT_Y234C*	Y234C	0.00	0.013	4.5
RM699	R273H	0.42	0.35	140
CT265	R273H	0.55	0.275	79
UD203	R273H	0.83	0.67	252
RMSA51	R273H	1.17	1.53	257
RM333	R273H	1.48	1.7	722
CT310	R273H	2.94	3.56	1412
Sm-Es	R273H	3.04	2.69	1032
Me-Mg	R273H	5.65	5.98	1600
UD46	R273H	5.71	5.51	2099
WT_R273H†	R273H	0.00	0.03	8.7

* Fractional abundance percentage of the WT was calculated as the median value of 4 WT cases;

† Fractional abundance percentage of the WT was calculated as the median value of 7 WT cases;

ddPCR Fractional Abundance and positive droplets according to ddCPR software.

Table S5. Clonal evolution of *TP53* mutations in *TP53* mutated cases

Case	*TP53* Mutation Coding*	Variant allele frequency Timepoint 1[†]	Variant allele frequency Timepoint 2[‡]	Relapsed yes/no	Mutation positive selected on ib	*BTK/PLCG2* Mutation Coding*	Variant allele frequency
CT265	c.701A>G,p.Y234C	80.62	83.21	yes		c.1442G>C,p.C481S	30.5
CT265	c.742C>T,p.R248W	0.83	0.00			c.1441T>C,p.C481R	11.3
CT265	c.818G>A,p.R273H	0.54	0.00				
CT265	c.746G>A,p.R249K	0.00	0.52				
CT265	c.880G>T,p.E294*	0.00	3.42				
CT280	c.1024C>T,p.R342*	53.07	86.97	yes	yes	wt	
CT280	c.743G>A,p.R248Q	12.59	0.00				
CT38	c.548C>G,p.S183*	19.9	0.00	yes		c.1442G>C,p.C481S	72.3
CT38	c.560-2A>T,p.?	7.69	0.97				
CT38	c.488A>G,p.Y163C	6.69	0.73				
CT38	c.701A>G,p.Y234C	6.31	0.00				
CT38	c.375+1G>T,p.?	3.61	0.00				
CT38	c.701A>C,p.Y234S	3.31	2.06				
CT38	c.470_475del,p.V157_R158del	2.7	0.33				
RM152	c.842A>T,p.D281V	66.17	87.62	yes	yes	wt	
RM152	c.742C>T,p.R248W	5.43	2.24				
RM152	c.641A>G,p.H214R	1.2	0.00				
RM699	c.587G>A,p.R196Q	43.71	3.76	yes		wt	
RM699	c.722C>A,p.S241Y	4.56	24.36				
RM699	c.832C>A,p.P278T	1.68	55.51		yes		
RM699	c.584T>C,p.I195T	0.89	1.76				
RM699	c.742C>T,p.R248W	0.88	1.20				
RM699	c.823T>C,p.C275R	0.58	0.46				
RM699	c.818G>A,p.R273H	0.42	3.30				
RM699	c.707A>G,p.Y236C	0.00	3.54				
RM699	c.830G>T,p.C277F	0.00	1.46				
RM699	c.842A>G,p.D281G	0.00	0.60				
RM767	c.524G>A,p.R175H	75.12	99.18	yes	yes	c.1441T>C,p.C481R	45.8
RM767						c.1441T>A,p.C481S	2.4
RM767						c.1442G>C,p.C481S	26.4
RM767						c.1442G>A,p.C481Y	13.8
RMGEM162	c.1031T>G,p.L344R	15.43	0.68	yes		wt	
RMGEM162	c.681dup,p.D228*	14.61	4.78				
RMGEM162	c.733G>A,p.G245S	9.92	30.88		yes		
RMGEM162	c.395A>G,p.K132R	0.9	0.93				
RMGEM162	c.395A>C,p.K132T	0.00	0.44				
RMGEM162	c.388_414del,p.L130_A138del	0.00	1.03				
RMGEM162	c.489C>G,p.Y163*	0.00	0.53				
RMGEM162	c.489C>G,p.Y163*	0.00	0.58				
RMGEM162	c.673-2A>G,p.?	0.00	1.50				
RMGEM162	c.673-2A>C,p.?	0.00	0.67				
RMGEM162	c.673-2A>T,p.?	0.00	0.40				
RMGEM162	c.716A>C,p.N239T	0.00	1.28				
RMGEM162	c.733G>C,p.G245R	0.00	0.91				
RMGEM162	c.746G>C,p.R249T	0.00	0.46				
RMGEM162	c.830G>T,p.C277F	0.00	0.37				
RMGEM162	c.875_876delinsC,p.K292Tfs*53	0.00	0.51				
RMGEM162	c.892G>T,p.E298*	0.00	0.85				
RMGEM162	c.919+2T>A,p.?	0.00	0.93				
RMGEM18	c.527G>T,p.C176F	80.39	82.45	yes		wt	
RMGEM49	c.653T>A,p.V218E	80.27	22.77	yes		c.1442G>C,p.C481S	7.5
RMGEM49						c.1442G>T,p.C481F	17.7
TS208	c.501_505del,p.Q167Hfs*12	6.04	29.57	yes	yes	c.1442G>C,p.C481S	10.6
TS33	c.733G>A,p.G245S	13.12	93	yes	yes	c.1442G>A,p.C481S	21.5
TS33	c.945_946del,p.Q317Afs*19	0.32	2.09			c.1442G>T,p.C481Y	1.8
TS33						c.1583T>G,p.L528W	31.8
TS33						c.2120C>T,p.S707F	1.1
TS33						c.2535A>T,p.L845F	3
TS33						c.3422T>A,p.M1141K	1.2
UD115	c.740A>T,p.N247I	91.7	89.37	yes		wt	
UD161	c.695T>C,p.I232T	82.09	82.37	yes		c.1441T>C,p.C481R	87.8
UD18	c.742C>T,p.R248W	6.55	0.00	yes		c.1442G>C,p.C481S	24.4
UD18	c.395A>T,p.K132M	2.03	4.94			c.2120C>T,p.S707F	2.1
UD18	c.817C>T,p.R273C	0.59	0.00			c.2535A>C,p.L845F	2.2
UD18	c.133_149del,p.L45fs*0	0.00	1.86				
UD216	c.743G>A,p.R248Q	41.14	43.56	yes		wt	
UD216	c.155_158delinsCAT,p.Q52Pfs*71	39.87	39.87				

UD74	c.817C>T,p.R273C	29.53	43.32	yes		c.1442G>C,p.C481S	79.7
UD74	c.743G>A,p.R248Q	2.81	0.00			c.1442G>T,p.C481F	1.9
UD74	c.830G>T,p.C277F	0.59	0.00				
UD74	c.747G>C,p.R249S	0.53	0.00				
UD74	c.469G>T,p.V157F	0.42	0.00				
UD74	c.742C>T,p.R248W	0.00	0.98				
CT304	c.626_627del,p.R209Kfs*6	71	88.75	no		wt	
CT304	c.843C>A,p.D281E	15	0.52				
CT60	c.733G>A,p.G245S	35.89	44.26	no		wt	
CT60	c.360del,p.K120Nfs*3	4.55	19.22				
CT60	c.734G>T,p.G245V	4.34	0.00				
CT60	c.848G>C,p.R283P	2.83	1.63				
CT60	c.641A>G,p.H214R	2.74	2.5				
CT60	c.673-2A>T,p.?	1.68	0.00				
RM113	c.814G>T,p.V272L	81.2	97.41	no		c.1442G>C,p.C481S	64.4
RM113						c.2535A>C,p.L845F	1
RM28	c.524G>A,p.R175H	26.18	51.68	no	yes	wt	
RM28	c.716A>G,p.N239S	25.25	48.63		yes		
RM312	c.470T>G,p.V157G	11.63	1.06	no		wt	
RM312	c.743G>A,p.R248Q	1.94	0.00				
RM312	c.733G>A,p.G245S	0.00	95.24		yes		
RM400	c.742C>G,p.R248G	12.12	10.94	no		wt	
RM400	c.841G>T,p.D281Y	8.19	9.93				
RM400	c.736A>G,p.M246V	1.79	2.13				
RM400	c.742C>T,p.R248W	0.83	0.00				
RM400	c.536A>T,p.H179L	0.73	0.68				
RM400	c.743G>A,p.R248Q	0.56	0.81				
RM400	c.706T>A,p.Y236N	0.3	0.4				
RM629	c.838A>G,p.R280G	88.67	50.98	no		c.1994G>A,p.R665Q	1.6
RM655	c.713G>A,p.C238Y	1.15	1.58	no		c.1441T>A,p.C481S	18.9
RM655						c.1442G>C,p.C481S	79.4
RM739	c.817C>T,p.R273C	38.57	17.69	no		wt	
RM739	c.743G>A,p.R248Q	3.52	4.01				
RM739	c.527G>A,p.C176Y	0.59	1.82				
RM739	c.757A>C,p.T253P	0.59	0.40				
RM739	c.826G>C,p.A276P	0.49	0.54				
RM739	c.818G>A,p.R273H	0.00	1.60				
RM739	c.830G>T,p.C277F	0.00	0.50				
RM739	c.856G>A,p.E286K	0.00	0.85				
RM76	c.517G>A,p.V173M	90	82.14	no		wt	
RM76	c.560-1G>A,p.?	0.00	8.93				
RMGEM38	c.734G>A,p.G245D	3.43	1.19	no		wt	
RMGEM38	c.472C>G,p.R158G	0.00	21.14		yes		
RMGEM38	c.524G>A,p.R175H	0.00	2.9				
RMGEM38	c.527G>A,p.C176Y	0.00	1.92				
RMGEM38	c.659A>G,p.Y220C	0.00	1.44				
RMGEM38	c.658T>C,p.Y220H	0.00	1.19				
RMGEM38	c.742C>T,p.R248W	0.00	1.93				
RMGEM38	c.742C>G,p.R248G	0.00	1.06				
RMGEM54	c.652_653insACC,p.V218delinsDL	3.81	8.96	no		wt	
RMPTV164	c.916C>T,p.R306*	78.97	92.48	no		wt	
RMPTV241	c.839G>C,p.R280T	38.81	37.31	no		wt	
RMPTV261	c.991del,p.Q331Rfs*14	2.54	2.01	no		wt	
RMPTV261	c.856G>A,p.E286K	2.31	0.44				
RMPTV261	c.393_395del,p.N131del	2.22	1.14				
RMPTV312	c.535C>G,p.H179D	53.33	0.00	no		c.1441T>A,p.C481S	1.2
RMPTV312	c.722C>T,p.S241F	43.63	7.32			c.1442G>C,p.C481S	13.6
RMPTV441	c.844C>T,p.R282W	30.62	0.00	no		wt	
RMPTV472	c.981T>G,p.Y327*	84.09	58.03	no		wt	
RMPTV472	c.990del,p.Q331Rfs*14	82.7	57.49				
RMPTV51	c.304del,p.T102Pfs*21	83.96	88	no		wt	
UD141	c.444_448del,p.S149Tfs*30	13.38	6.6	no		wt	
UD141	c.742C>T,p.R248W	1.3	0.55				
UD141	c.743G>A,p.R248Q	1.1	0.00				
UD141	c.527G>A,p.C176Y	0.68	0.00				
UD141	c.422G>A,p.C141Y	0.00	9.2				
UD141	c.578A>G,p.H193R	0.00	0.73				
UD150	c.584T>A,p.I195N	1.85	3.09	no		wt	
UD185	c.740A>T,p.N247I	96.79	16.52	no		wt	
UD185	c.151del,p.E51Nfs*72	2.12	0.96				

* According to Human Genome Variation Society (HGVS) nomenclature. https://www.mutalyzer.nl/

† Collected before ibrutinib treatment

‡ Collected at ibrutinib end for relapsed cases, or during ibrutinib treatment for non-relapsed cases

Mutation coding refferring to *PLCG2* are highlighted in grey.
